# Bis[(diphenyl­phosphanylmeth­yl)diphenyl­phosphane sulfide-κ^2^ *P*,*S*]copper(I) hexa­fluoridophosphate

**DOI:** 10.1107/S160053681202346X

**Published:** 2012-05-31

**Authors:** Jing-Jing Zhang, Feng Hu, Tai-Ke Duan, Qun Chen, Qian-Feng Zhang

**Affiliations:** a Institute of Molecular Engineering and Applied Chemistry, Anhui University of Technology, Ma’anshan, Anhui 243002, People’s Republic of China; b Department of Applied Chemistry, School of Petrochemical Engineering, Changzhou University, Jiangsu 213164, People’s Republic of China

## Abstract

In the title compound, [Cu(C_25_H_22_P_2_S)_2_]PF_6_, the Cu^I^ atom, lying on a twofold rotation axis, adopts a distorted tetra­hedral geometry. The (diphenyl­phosphanylmeth­yl)diphenyl­phos­phane sulfide ligand coordinates to the Cu^I^ atom through one S and one P atom, forming a stable five-membered chelate ring. The P atom of the PF_6_^−^ anion also lies on a twofold rotation axis.

## Related literature

For background to copper(I) phosphane compounds, see: Bownaker *et al.* (1995 ▶); Comba *et al.* (1999 ▶); Liaw *et al.* (2005 ▶); Lobana *et al.* (2009 ▶); Nicola *et al.* (2005 ▶); Zhang *et al.* (2005 ▶). For related structures, see: Bera *et al.* (1999 ▶); Sivasankar *et al.* (2004 ▶).
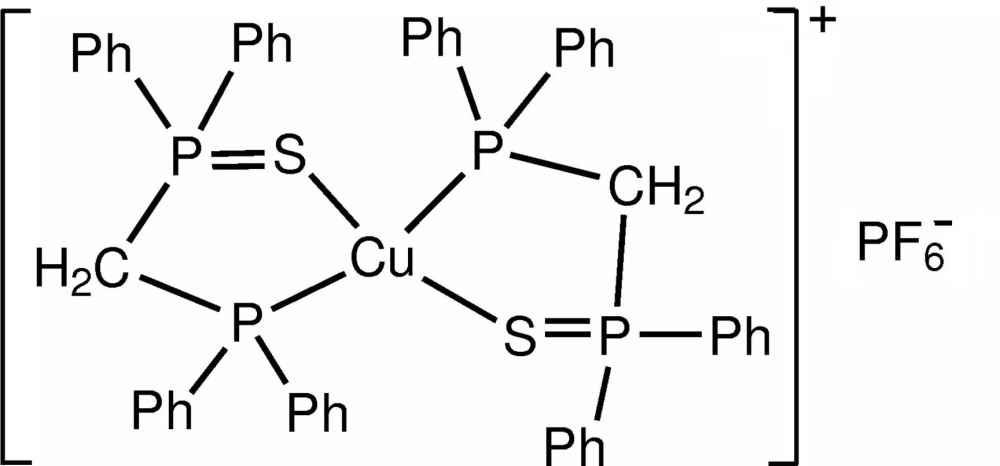


## Experimental

### 

#### Crystal data

[Cu(C_25_H_22_P_2_S)_2_]PF_6_
*M* *_r_* = 1041.39
Orthorhombic, 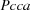

*a* = 20.73 (3) Å
*b* = 12.004 (18) Å
*c* = 19.83 (3) Å
*V* = 4935 (13) Å^3^
*Z* = 4
Mo *K*α radiation
μ = 0.75 mm^−1^
*T* = 296 K
0.26 × 0.22 × 0.17 mm

#### Data collection

Bruker APEXII CCD diffractometer
Absorption correction: multi-scan (*SADABS*; Sheldrick, 1996) ▶ *T*_min_ = 0.830, *T*_max_ = 0.884
27988 measured reflections
5535 independent reflections
3424 reflections with *I* > 2σ(*I*)
*R*_int_ = 0.070

#### Refinement

*R*[*F* ^2^ > 2σ(*F* ^2^)] = 0.053
*wR*(*F* ^2^) = 0.152
*S* = 1.03
5535 reflections
290 parameters
H-atom parameters constrained
Δρ_max_ = 0.41 e Å^−3^
Δρ_min_ = −0.55 e Å^−3^

### 

Data collection: *APEX2* (Bruker, 2007 ▶); cell refinement: *SAINT* (Bruker, 2007 ▶); data reduction: *SAINT*; program(s) used to solve structure: *SHELXS97* (Sheldrick, 2008 ▶); program(s) used to refine structure: *SHELXL97* (Sheldrick, 2008 ▶); molecular graphics: *SHELXTL* (Sheldrick, 2008 ▶); software used to prepare material for publication: *SHELXTL*.

## Supplementary Material

Crystal structure: contains datablock(s) I, global. DOI: 10.1107/S160053681202346X/hy2551sup1.cif

Structure factors: contains datablock(s) I. DOI: 10.1107/S160053681202346X/hy2551Isup2.hkl

Additional supplementary materials:  crystallographic information; 3D view; checkCIF report

## Figures and Tables

**Table 1:** Selected bond lengths (Å)

Cu1—P2	2.300 (3)
Cu1—S1	2.411 (3)

**Fig. 1.**
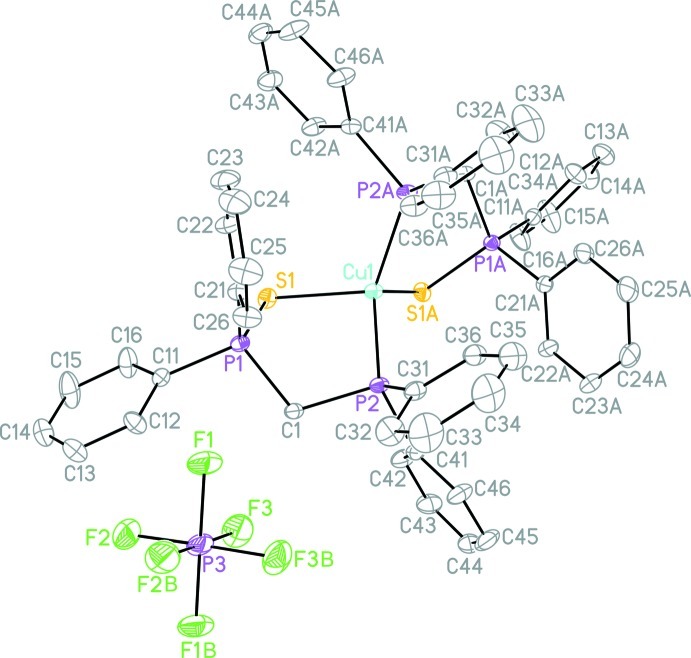
Molecular structure of the title compound, with displacement ellipsoids at the 50% probability level. [Symmetry codes: (A) 1/2-x, 1-y, z; (B) 1-x, y, 1/2-z.]

## supplementary crystallographic information

### Comment

The chemistry of copper(I) remains on the forefront in binding to soft Lewis bases such as phosphorous and sulfur donors (Liaw *et al.*, 2005; Zhang *et al.*, 2005). For examples, there are a number of published studies of structures that involve copper(I) complexes with phosphane ligands in variable copper(I)-to-ligand ratios (Bownaker *et al.*, 1995; Comba *et al.*, 1999). Mononuclear and dinuclear phosphane-copper(I) complexes with coordinated and bridging halide anions and phosphane ligands in various coordination modes have been well isolated and structurally characterized (Lobana *et al.*, 2009). Quite a few copper(I) complexes with mixed phosphane and sulfide ligands have been synthesized and structurally measured by X-ray crystallography (Lobana *et al.*, 2009; Nicola *et al.*, 2005). Although adducts of bis(diphenylphosphanyl)methane (dppm), structurally defined complexes of the form CuX:dppm (1:1) (X = Cl, Br, I, CN, SCN), have been well documented (Nicola *et al.*, 2005), only one example of mononuclear copper(I) complex with (diphenylphosphanylmethyl)diphenylphosphane sulfide (dppmS) that involves in oxidation of one phosphorus atom of the dppm ligand to P=S moiety has been reported (Sivasankar *et al.*, 2004). The second example of mononuclear copper(I) complex with dppmS ligands is described in this paper.

The title compound consists of a cationic [Cu(dppmS)_2_]^+^ unit and a PF_6_^-^ anion (Fig. 1). The dppmS ligand coordinates to the Cu^I^ atom with one S and one P atoms, forming a stable five-membered chelating ring. The coordinating environment around the Cu^I^ atom is distorted tetrahedral. The Cu—P bond length (Table 1) is similar to those found in [Cu(dppmS)_2_][ClO_4_] (Sivasankar *et al.*, 2004) and in the copper(I)-dppm complexes (Bera *et al.*, 1999). The Cu—S bond length of 2.411 (3) Å agrees well with that of 2.395 (3) Å in [Cu(dppmS)_2_][ClO_4_] (Sivasankar *et al.*, 2004). The P—Cu—P bond angle of 127.60 (11)° is obviously larger than the S—Cu—S bond angle of 101.63 (11)°, due to the bulky PPh_2_ moiety directly binding to the Cu atom. The P—Cu—S bond angle of 97.37 (6)° in the five-membered ring of the dppmS ligand is more acute than that of 115.45 (5)° between two dppmS ligands. The PF_6_^-^ anion has its expected structure as well as normal distances and angles.

### Experimental

To a solution of [Cu(CH_3_CN)_4_][PF_6_] (373 mg, 1.0 mmol) in CH_3_CN (10 ml) was added with a dppm (796 mg, 2.0 mmol) solution in CH_2_Cl_2_ (5 ml) and S_8_ powder (64 mg, 2.0 mmol). After the mixture was stirred for 4 h at room temperature, the colorless solution with a little brown precipitate was obtained. After filtration, colorless block crystals were formed by slow evaporation of the filtrate at room temperature in three days. Analysis, calculated for C_50_H_44_CuF_6_P_5_S_2_: C 57.66, H 4.26%; found: C 57.53, H 4.23%.

### Refinement

H atoms were positioned geometrically and refined as riding atoms, with C—H = 0.93 (aromatic) and 0.97 (CH_2_) Å and with *U*_iso_(H) = 1.2*U*_eq_(C).

### Crystal data

**Table:**

[Cu(C_25_H_22_P_2_S)_2_]PF_6_	*F*(000) = 2136
*M**_r_* = 1041.39	*D*_x_ = 1.402 Mg m^−^^3^
Orthorhombic, *P**c**c**a*	Mo *K*α radiation, λ = 0.71073 Å
Hall symbol: -P 2a 2ac	Cell parameters from 2469 reflections
*a* = 20.73 (3) Å	θ = 1.0–24.6°
*b* = 12.004 (18) Å	µ = 0.75 mm^−^^1^
*c* = 19.83 (3) Å	*T* = 296 K
*V* = 4935 (13) Å^3^	Block, colorless
*Z* = 4	0.26 × 0.22 × 0.17 mm

### Data collection

**Table:**

Bruker APEXII CCD diffractometer	5535 independent reflections
Radiation source: fine-focus sealed tube	3424 reflections with *I* > 2σ(*I*)
Graphite monochromator	*R*_int_ = 0.070
φ and ω scans	θ_max_ = 27.3°, θ_min_ = 2.3°
Absorption correction: multi-scan (*SADABS*; Sheldrick, 1996)	*h* = −26→26
*T*_min_ = 0.830, *T*_max_ = 0.884	*k* = −15→7
27988 measured reflections	*l* = −25→25

### Refinement

**Table:**

Refinement on *F*^2^	Primary atom site location: structure-invariant direct methods
Least-squares matrix: full	Secondary atom site location: difference Fourier map
*R*[*F*^2^ > 2σ(*F*^2^)] = 0.053	Hydrogen site location: inferred from neighbouring sites
*wR*(*F*^2^) = 0.152	H-atom parameters constrained
*S* = 1.03	*w* = 1/[σ^2^(*F*_o_^2^) + (0.0663*P*)^2^ + 1.7941*P*] where *P* = (*F*_o_^2^ + 2*F*_c_^2^)/3
5535 reflections	(Δ/σ)_max_ < 0.001
290 parameters	Δρ_max_ = 0.41 e Å^−^^3^
0 restraints	Δρ_min_ = −0.55 e Å^−^^3^

### Special details

**Table:**

Geometry. All esds (except the esd in the dihedral angle between two l.s. planes) are estimated using the full covariance matrix. The cell esds are taken into account individually in the estimation of esds in distances, angles and torsion angles; correlations between esds in cell parameters are only used when they are defined by crystal symmetry. An approximate (isotropic) treatment of cell esds is used for estimating esds involving l.s. planes.
Refinement. Refinement of F^2^ against ALL reflections. The weighted R-factor wR and goodness of fit S are based on F^2^, conventional R-factors R are based on F, with F set to zero for negative F^2^. The threshold expression of F^2^ > 2sigma(F^2^) is used only for calculating R-factors(gt) etc. and is not relevant to the choice of reflections for refinement. R-factors based on F^2^ are statistically about twice as large as those based on F, and R- factors based on ALL data will be even larger.

### Fractional atomic coordinates and isotropic or equivalent isotropic displacement parameters (Å^2^)

**Table:**

	*x*	*y*	*z*	*U*_iso_*/*U*_eq_	
Cu1	0.2500	0.5000	0.08447 (3)	0.0570 (2)	
S1	0.25626 (4)	0.34472 (9)	0.00764 (4)	0.0673 (3)	
P1	0.30722 (4)	0.25355 (8)	0.07262 (4)	0.0545 (2)	
P2	0.34846 (4)	0.47449 (8)	0.13570 (4)	0.0571 (3)	
P3	0.5000	0.08241 (18)	0.2500	0.1137 (7)	
F1	0.42534 (14)	0.0827 (3)	0.2603 (2)	0.1813 (17)	
F2	0.49237 (19)	−0.0099 (3)	0.1934 (2)	0.1708 (17)	
F3	0.49046 (19)	0.1782 (3)	0.1953 (2)	0.1685 (15)	
C1	0.37539 (13)	0.3349 (3)	0.10528 (16)	0.0591 (8)	
H1A	0.4073	0.3444	0.0699	0.071*	
H1B	0.3955	0.2947	0.1421	0.071*	
C11	0.34201 (15)	0.1313 (3)	0.03184 (16)	0.0602 (8)	
C12	0.3884 (2)	0.0687 (4)	0.0633 (2)	0.0860 (13)	
H12	0.4014	0.0875	0.1068	0.103*	
C13	0.4165 (2)	−0.0224 (4)	0.0314 (3)	0.1030 (15)	
H13	0.4476	−0.0642	0.0538	0.124*	
C14	0.3990 (3)	−0.0504 (5)	−0.0318 (3)	0.1114 (16)	
H14	0.4176	−0.1117	−0.0529	0.134*	
C15	0.3543 (4)	0.0107 (5)	−0.0643 (3)	0.157 (3)	
H15	0.3424	−0.0085	−0.1080	0.188*	
C16	0.3256 (3)	0.1031 (4)	−0.0330 (2)	0.1212 (19)	
H16	0.2953	0.1454	−0.0563	0.145*	
C21	0.25957 (15)	0.2067 (3)	0.14369 (17)	0.0640 (9)	
C22	0.19323 (17)	0.1912 (4)	0.1354 (2)	0.0852 (12)	
H22	0.1741	0.2056	0.0939	0.102*	
C23	0.1559 (2)	0.1545 (4)	0.1891 (3)	0.1142 (17)	
H23	0.1119	0.1432	0.1832	0.137*	
C24	0.1833 (3)	0.1347 (5)	0.2504 (3)	0.122 (2)	
H24	0.1577	0.1117	0.2863	0.146*	
C25	0.2485 (3)	0.1485 (5)	0.2599 (2)	0.120 (2)	
H25	0.2667	0.1338	0.3018	0.144*	
C26	0.2877 (2)	0.1846 (4)	0.20634 (18)	0.0879 (13)	
H26	0.3318	0.1938	0.2125	0.105*	
C31	0.35556 (17)	0.4728 (4)	0.22775 (17)	0.0714 (10)	
C32	0.3949 (3)	0.4004 (5)	0.2639 (2)	0.1215 (19)	
H32	0.4197	0.3472	0.2418	0.146*	
C33	0.3962 (4)	0.4097 (8)	0.3352 (3)	0.175 (3)	
H33	0.4208	0.3609	0.3609	0.211*	
C34	0.3596 (4)	0.4936 (8)	0.3663 (3)	0.165 (3)	
H34	0.3600	0.4988	0.4131	0.198*	
C35	0.3239 (3)	0.5671 (6)	0.3308 (2)	0.122 (2)	
H35	0.3017	0.6239	0.3527	0.147*	
C36	0.32075 (18)	0.5565 (4)	0.26102 (18)	0.0862 (13)	
H36	0.2953	0.6055	0.2362	0.103*	
C41	0.41620 (14)	0.5644 (3)	0.10968 (17)	0.0621 (9)	
C42	0.42341 (17)	0.5905 (4)	0.0432 (2)	0.0885 (13)	
H42	0.3951	0.5599	0.0119	0.106*	
C43	0.4723 (2)	0.6623 (5)	0.0204 (3)	0.1042 (16)	
H43	0.4765	0.6773	−0.0254	0.125*	
C44	0.5129 (2)	0.7090 (4)	0.0646 (3)	0.1016 (15)	
H44	0.5459	0.7550	0.0494	0.122*	
C45	0.5059 (2)	0.6893 (5)	0.1319 (3)	0.1221 (19)	
H45	0.5330	0.7245	0.1626	0.146*	
C46	0.45783 (19)	0.6157 (5)	0.1550 (2)	0.1002 (16)	
H46	0.4539	0.6013	0.2009	0.120*	

### Atomic displacement parameters (Å^2^)

**Table:**

	*U*^11^	*U*^22^	*U*^33^	*U*^12^	*U*^13^	*U*^23^
Cu1	0.0448 (3)	0.0748 (5)	0.0513 (3)	−0.0022 (3)	0.000	0.000
S1	0.0792 (6)	0.0684 (7)	0.0544 (5)	−0.0009 (5)	−0.0183 (4)	0.0036 (4)
P1	0.0491 (4)	0.0655 (6)	0.0490 (4)	−0.0057 (4)	−0.0055 (3)	0.0063 (4)
P2	0.0434 (4)	0.0800 (7)	0.0480 (4)	−0.0053 (4)	−0.0010 (3)	−0.0079 (4)
P3	0.0781 (10)	0.1068 (16)	0.1562 (19)	0.000	−0.0655 (11)	0.000
F1	0.0818 (19)	0.185 (4)	0.278 (5)	0.002 (2)	−0.058 (2)	−0.012 (3)
F2	0.152 (3)	0.140 (3)	0.220 (4)	−0.010 (2)	−0.064 (3)	−0.045 (3)
F3	0.186 (3)	0.137 (3)	0.182 (3)	0.008 (2)	−0.090 (3)	0.026 (3)
C1	0.0446 (15)	0.073 (2)	0.0595 (18)	−0.0040 (15)	−0.0001 (13)	−0.0038 (17)
C11	0.0624 (18)	0.058 (2)	0.0598 (19)	−0.0064 (16)	−0.0009 (15)	0.0016 (16)
C12	0.088 (3)	0.102 (4)	0.068 (2)	0.028 (2)	−0.0006 (19)	−0.007 (2)
C13	0.095 (3)	0.112 (4)	0.102 (4)	0.036 (3)	0.005 (3)	−0.002 (3)
C14	0.143 (5)	0.085 (4)	0.106 (4)	0.021 (3)	0.017 (3)	−0.010 (3)
C15	0.270 (9)	0.107 (5)	0.094 (4)	0.055 (5)	−0.060 (5)	−0.035 (3)
C16	0.181 (5)	0.083 (4)	0.100 (3)	0.031 (3)	−0.067 (3)	−0.019 (3)
C21	0.0567 (18)	0.072 (3)	0.0632 (19)	−0.0018 (16)	0.0013 (14)	0.0155 (18)
C22	0.057 (2)	0.098 (3)	0.100 (3)	−0.015 (2)	0.0021 (19)	0.022 (3)
C23	0.071 (3)	0.118 (4)	0.154 (5)	−0.021 (3)	0.021 (3)	0.042 (4)
C24	0.113 (4)	0.132 (5)	0.121 (4)	0.001 (3)	0.045 (3)	0.055 (4)
C25	0.121 (4)	0.154 (6)	0.085 (3)	0.015 (4)	0.013 (3)	0.058 (3)
C26	0.075 (2)	0.121 (4)	0.068 (2)	0.008 (2)	0.0031 (19)	0.036 (2)
C31	0.0624 (19)	0.101 (3)	0.0513 (18)	−0.010 (2)	−0.0058 (16)	−0.0060 (19)
C32	0.143 (4)	0.154 (5)	0.068 (3)	0.013 (4)	−0.036 (3)	−0.001 (3)
C33	0.244 (8)	0.206 (9)	0.076 (4)	0.028 (7)	−0.068 (5)	0.011 (4)
C34	0.229 (8)	0.217 (9)	0.049 (3)	0.004 (6)	−0.013 (4)	−0.013 (4)
C35	0.126 (4)	0.182 (6)	0.060 (3)	−0.004 (4)	0.005 (3)	−0.031 (3)
C36	0.074 (2)	0.125 (4)	0.060 (2)	−0.006 (2)	0.0007 (17)	−0.021 (2)
C41	0.0447 (15)	0.075 (3)	0.066 (2)	0.0004 (16)	0.0017 (14)	−0.0104 (18)
C42	0.070 (2)	0.127 (4)	0.068 (2)	−0.026 (2)	0.0032 (18)	−0.001 (2)
C43	0.079 (3)	0.139 (5)	0.095 (3)	−0.015 (3)	0.021 (2)	0.010 (3)
C44	0.071 (3)	0.101 (4)	0.132 (4)	−0.018 (2)	0.031 (3)	−0.005 (3)
C45	0.083 (3)	0.146 (5)	0.137 (5)	−0.055 (3)	−0.002 (3)	−0.034 (4)
C46	0.077 (2)	0.143 (5)	0.081 (3)	−0.043 (3)	−0.005 (2)	−0.013 (3)

### Geometric parameters (Å, º)

**Table:**

Cu1—P2	2.300 (3)	C22—H22	0.9300
Cu1—S1	2.411 (3)	C23—C24	1.363 (7)
S1—P1	1.993 (2)	C23—H23	0.9300
P1—C21	1.810 (4)	C24—C25	1.374 (7)
P1—C11	1.824 (4)	C24—H24	0.9300
P1—C1	1.836 (4)	C25—C26	1.405 (6)
P2—C31	1.831 (4)	C25—H25	0.9300
P2—C41	1.845 (4)	C26—H26	0.9300
P2—C1	1.866 (4)	C31—C32	1.390 (6)
P3—F1	1.561 (4)	C31—C36	1.403 (6)
P3—F1^i^	1.561 (4)	C32—C33	1.418 (7)
P3—F2	1.585 (4)	C32—H32	0.9300
P3—F2^i^	1.585 (4)	C33—C34	1.403 (10)
P3—F3^i^	1.593 (4)	C33—H33	0.9300
P3—F3	1.593 (4)	C34—C35	1.351 (9)
C1—H1A	0.9700	C34—H34	0.9300
C1—H1B	0.9700	C35—C36	1.391 (6)
C11—C12	1.371 (5)	C35—H35	0.9300
C11—C16	1.373 (6)	C36—H36	0.9300
C12—C13	1.392 (6)	C41—C42	1.364 (5)
C12—H12	0.9300	C41—C46	1.390 (5)
C13—C14	1.347 (7)	C42—C43	1.405 (6)
C13—H13	0.9300	C42—H42	0.9300
C14—C15	1.345 (8)	C43—C44	1.337 (7)
C14—H14	0.9300	C43—H43	0.9300
C15—C16	1.404 (7)	C44—C45	1.361 (7)
C15—H15	0.9300	C44—H44	0.9300
C16—H16	0.9300	C45—C46	1.409 (6)
C21—C26	1.397 (5)	C45—H45	0.9300
C21—C22	1.397 (5)	C46—H46	0.9300
C22—C23	1.388 (6)		
			
P2^ii^—Cu1—P2	127.60 (11)	C11—C16—H16	119.9
P2^ii^—Cu1—S1	115.45 (5)	C15—C16—H16	119.9
P2—Cu1—S1	97.37 (6)	C26—C21—C22	119.3 (3)
P2^ii^—Cu1—S1^ii^	97.37 (6)	C26—C21—P1	121.5 (3)
P2—Cu1—S1^ii^	115.45 (5)	C22—C21—P1	119.1 (3)
S1—Cu1—S1^ii^	101.63 (13)	C23—C22—C21	120.0 (4)
P1—S1—Cu1	92.55 (4)	C23—C22—H22	120.0
C21—P1—C11	108.11 (19)	C21—C22—H22	120.0
C21—P1—C1	108.11 (18)	C24—C23—C22	120.5 (4)
C11—P1—C1	106.27 (17)	C24—C23—H23	119.8
C21—P1—S1	112.60 (15)	C22—C23—H23	119.8
C11—P1—S1	111.40 (15)	C23—C24—C25	120.7 (4)
C1—P1—S1	110.10 (15)	C23—C24—H24	119.6
C31—P2—C41	102.94 (16)	C25—C24—H24	119.6
C31—P2—C1	106.73 (18)	C24—C25—C26	120.2 (5)
C41—P2—C1	101.95 (17)	C24—C25—H25	119.9
C31—P2—Cu1	120.86 (12)	C26—C25—H25	119.9
C41—P2—Cu1	118.30 (14)	C21—C26—C25	119.3 (4)
C1—P2—Cu1	104.02 (10)	C21—C26—H26	120.4
F1—P3—F1^i^	179.7 (4)	C25—C26—H26	120.4
F1—P3—F2	89.8 (2)	C32—C31—C36	120.5 (4)
F1^i^—P3—F2	90.4 (2)	C32—C31—P2	124.6 (3)
F1—P3—F2^i^	90.4 (2)	C36—C31—P2	114.8 (3)
F1^i^—P3—F2^i^	89.8 (2)	C31—C32—C33	118.4 (6)
F2—P3—F2^i^	91.3 (4)	C31—C32—H32	120.8
F1—P3—F3^i^	91.8 (2)	C33—C32—H32	120.8
F1^i^—P3—F3^i^	88.0 (2)	C34—C33—C32	118.9 (6)
F2—P3—F3^i^	177.5 (3)	C34—C33—H33	120.5
F2^i^—P3—F3^i^	90.6 (3)	C32—C33—H33	120.5
F1—P3—F3	88.0 (2)	C35—C34—C33	122.5 (5)
F1^i^—P3—F3	91.8 (2)	C35—C34—H34	118.8
F2—P3—F3	90.6 (3)	C33—C34—H34	118.8
F2^i^—P3—F3	177.5 (3)	C34—C35—C36	118.9 (6)
F3^i^—P3—F3	87.6 (3)	C34—C35—H35	120.5
P1—C1—P2	111.18 (17)	C36—C35—H35	120.5
P1—C1—H1A	109.4	C35—C36—C31	120.6 (5)
P2—C1—H1A	109.4	C35—C36—H36	119.7
P1—C1—H1B	109.4	C31—C36—H36	119.7
P2—C1—H1B	109.4	C42—C41—C46	117.1 (4)
H1A—C1—H1B	108.0	C42—C41—P2	119.2 (3)
C12—C11—C16	117.8 (4)	C46—C41—P2	123.4 (3)
C12—C11—P1	121.1 (3)	C41—C42—C43	122.0 (4)
C16—C11—P1	121.0 (3)	C41—C42—H42	119.0
C11—C12—C13	121.1 (4)	C43—C42—H42	119.0
C11—C12—H12	119.4	C44—C43—C42	120.1 (5)
C13—C12—H12	119.4	C44—C43—H43	120.0
C14—C13—C12	120.4 (5)	C42—C43—H43	120.0
C14—C13—H13	119.8	C43—C44—C45	120.1 (4)
C12—C13—H13	119.8	C43—C44—H44	119.9
C15—C14—C13	119.7 (5)	C45—C44—H44	119.9
C15—C14—H14	120.2	C44—C45—C46	120.2 (4)
C13—C14—H14	120.2	C44—C45—H45	119.9
C14—C15—C16	120.8 (5)	C46—C45—H45	119.9
C14—C15—H15	119.6	C41—C46—C45	120.4 (4)
C16—C15—H15	119.6	C41—C46—H46	119.8
C11—C16—C15	120.2 (5)	C45—C46—H46	119.8

Symmetry codes: (i) −*x*+1, *y*, −*z*+1/2; (ii) −*x*+1/2, −*y*+1, *z*.
